# Obstetric Mentor

**Published:** 1884-03

**Authors:** 


AN OBSTETRIC MENTOR. A hand-book of the Homœopathic Treatment required during Pregnancy, Parturition, and the Puerperal season. Pp. 219. By Clarence M. Conant, M. D. New York: A. L. Chatterton Publishing Company. 1884.

The title-page of Dr. Conant's little volume, as given above, shows the subjects upon which it treats. They are those upon which we all, at times, need the best advice. This Dr. Conant's book furnishes. This is one of the books that every physician who practices *Homœopathy* should possess.

